# From Free‐Radical to Radical‐Free: A Paradigm Shift in Light‐Mediated Biofabrication

**DOI:** 10.1002/advs.202205302

**Published:** 2023-01-25

**Authors:** Riccardo Rizzo, Nika Petelinšek, Angela Bonato, Marcy Zenobi‐Wong

**Affiliations:** ^1^ Tissue Engineering + Biofabrication Laboratory Department of Health Sciences & Technology ETH Zürich Otto‐Stern‐Weg 7 Zürich 8093 Switzerland

**Keywords:** bioprinting, free‐radical, photochemistry, photoclick, reactive oxygen species, thiol‐ene, two‐photon

## Abstract

In recent years, the development of novel photocrosslinking strategies and photoactivatable materials has stimulated widespread use of light‐mediated biofabrication techniques. However, despite great progress toward more efficient and biocompatible photochemical strategies, current photoresins still rely on photoinitiators (PIs) producing radical‐initiating species to trigger the so‐called free‐radical crosslinking/polymerization. In the context of bioprinting, where cells are encapsulated in the bioink, the presence of radicals raises concerns of potential cytotoxicity. In this work, a universal, radical‐free (RF) photocrosslinking strategy to be used for light‐based technologies is presented. Leveraging RF uncaging mechanisms and Michael addition, cell‐laden constructs are photocrosslinked by means of one‐ and two‐photon excitation with high biocompatibility. A hydrophilic coumarin‐based group is used to cage a universal RF photocrosslinker based on 4‐arm‐PEG‐thiol (PEG4SH). Upon light exposure, thiols are uncaged and react with an alkene counterpart to form a hydrogel. RF photocrosslinker is shown to be highly stable, enabling potential for off‐the‐shelf products. While PI‐based systems cause a strong upregulation of reactive oxygen species (ROS)‐associated genes, ROS are not detected in RF photoresins. Finally, optimized RF photoresin is successfully exploited for high resolution two‐photon stereolithography (2P‐SL) using remarkably low polymer concentration (<1.5%), paving the way for a shift toward radical‐free light‐based bioprinting.

## Graphical abstract

Photoinitiators producing radicals are commonly used to trigger free‐radical crosslinking, raising concerns of potential cytotoxicity. Here, a universal, radical‐free (RF) photocrosslinking strategy is presented. Leveraging RF uncaging and Michael‐addition, cell‐laden constructs are generated by means of one‐ and two‐photon irradiation. With high biocompatibility and negligible reactive oxygen species (ROS), RF‐crosslinking ushers in a new era for light‐mediated bioprinting.

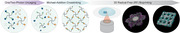

## 1 Introduction

Light can serve as a remote, contactless trigger to drive a wide range of photochemical reactions. In tissue engineering, this powerful tool is used to form or break chemical bonds,^[^ ^1^, ^2^ ^]^ playing an increasingly important role in the field of 3D bioprinting.^[^ ^3^, ^4^ ^]^ Photoactivated materials have been introduced in traditional bioprinting methods such as extrusion printing, or for technologies that have specifically been designed for light‐triggered processes, such as one‐photon stereolithography (digital light processing, lase scanning Stereolithography),^[^ ^3^, ^5^, ^6^, ^7^ ^]^ two‐photon stereolithography (2P‐SL),^[^ ^3^, ^8^, ^9^ ^]^ and more recently volumetric bioprinting (VP).^[^ ^10^, ^11^, ^12^ ^]^ Despite major advances in the design of highly reactive and biocompatible photoresins, light‐based printing still relies on free radicals to trigger the crosslinking mechanism. When cells are embedded in photoactivated hydrogels, the presence of free‐radicals and, consequently, reactive oxygen species (ROS), raises concerns of potential cytotoxicity.^[^ ^13^, ^14^, ^15^, ^16^, ^17^ ^]^

**Figure 1:**
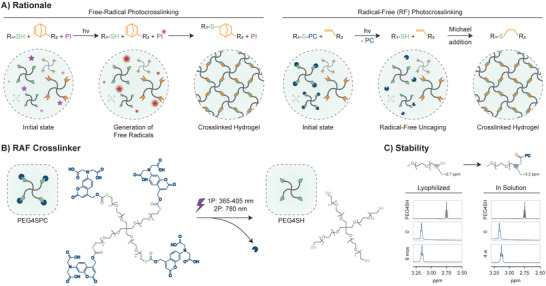
A) Rationale of the work. Thiol‐norbornene step‐growth chemistry is illustrated as an example of free‐radical photocrosslinking (left). Upon light absorption, the photoinitiator (PI) radicals (red) drive the formation of a crosslinked network. In the case of free‐radical (RF) approach (right), upon light absorption, the photocages (PC, blue) are removed in a radical‐free fashion. This exposes thiols (green) to Michael addition with ‐ene (orange) functionalized polymers, resulting in the formation of a crosslinked hydrogel. B) Chemical structure of RF crosslinker PEG4SPC. PEG4SH is caged with PC via the formation of a thiocarbonate bond. Upon one‐ or two‐photon excitation, PC is removed and PEG4SH is re‐formed. C) Stability study of RF crosslinker with ^1^H‐NMR. Shift of terminal methylene in uncaged (highlighted in grey) and caged form (highlighted in blue), from ≈2.7 ppm to ≈3.2 ppm, was used to identify uncaging degree. PEG4SPC was found to be perfectly stable (no detectable uncaging) when kept in the dark in lyophilized form (stored at −20 °C) for up to 6 months and in solution (D_2_O) for up to 1 month (stored at 25 °C).

Common photoresin formulations contain a photoinitiator (PI) species that absorbs light and then undergoes a photochemical reaction that generates unpaired valence electrons (radicals) (**Figure** **1**A). PI radicals then initiate and propagate the crosslinking process that can occur via chain‐growth (i.e., for acryloyl and methacryloyl groups) or step‐growth (i.e., for thiol‐norbornene photoclick chemistry) reactions. Although more efficient, water‐soluble and biocompatible PIs such as lithium phenyl‐2,4,6‐trimethylbenzoylphosphinate (LAP) have been introduced in recent years, radical species remain an intrinsic disadvantage for biocompatibility.^[^ ^13^, ^14^, ^15^, ^16^, ^17^ ^]^ Most light‐based bioprinting studies lack in‐depth analysis on the deleterious effect of light and radicals. However, some studies in this direction made clear that mild near‐UV irradiation (≈1–20 mW cm^−2^) itself does not lead to cell damage,^[^ ^16^ ^]^ but it is instead the generation of radicals that leads to negative downstream effects. Although not been studied in terms of radicals production and their impact on cellular response, some PI‐free photo‐crosslinking/polymerization strategies such as cationic polymerization,^[^ ^18^, ^19^ ^]^ photodimerization of chromophores,^[^ ^20^, ^21^, ^22^ ^]^ tetrazole‐ene cycloaddition,^[^ ^23^ ^]^ and photoregulated hydrazone or imine formation^[^ ^24^, ^25^ ^]^ have been previously explored to generate polymeric networks. In addition to the limited literature on biofabrication performance and applications of these materials, they still largely rely on UV light (**≤**365 nm), can form reactive and potentially toxic byproducts (i.e., nitrosobenzaldehydes from nitrobenzyl moieties), and retain the photoabsorbing moiety bound to the polymeric network. For applications where, for example, delicate radical‐sensitive cells are used for biofabrication purposes, a simple and widely usable alternative approach is currently missing.

In this study, a universal radical‐free (RF) strategy is presented, showing the possibility of shifting the deep‐rooted paradigm of free‐radical photocrosslinking to radical‐free (RF) (Figure 1A). The RF method relies on a radical‐free photouncaging mechanism,^[^ ^26^ ^]^ and base‐catalyzed click reaction between thiols and electron‐deficient alkene groups (Michael addition).^[^ ^27^, ^28^ ^]^ Photocages (PC), also known as photoremovable protecting groups, are light‐sensitive molecules that temporally inactivate/mask active compounds or functional groups by being bound to them.^[^ ^26^ ^]^ As they can be released on demand via photolysis, this approach has been adopted in biological studies to have spatiotemporal control over cellular processes.^[^ ^26^, ^29^ ^]^ PCs have also found a role in tissue engineering, to gain spatiotemporal control over mechanical properties by introducing them in photodegradable linkers^[^ ^30^, ^31^, ^32^, ^33^ ^]^ or to liberate bioactive molecules or functional groups for patterning purposes.^[^ ^34^, ^35^, ^36^, ^37^, ^38^ ^]^ Unlike previous approaches, here we introduce a novel strategy in which a hydrophilic coumarin‐based PC is employed for (bio)fabrication by masking a thiolated crosslinker, thus providing spatiotemporal control over thiol‐Michael addition reaction. Upon light exposure, the PC is removed in a radical‐free fashion and thiols react with ‐ene modified polymers to form a hydrogel. Thiol‐Michael addition reaction has been widely used for many tissue engineering applications, due to its ability to proceed in a robust, click‐fashion under physiological conditions.^[^ ^27^, ^39^ ^]^

Starting from the synthesis and characterization of a universal RF crosslinker based on 4‐arm‐PEG‐thiol (PEG4SH), we demonstrated high stability of the product and wide tunability of reaction kinetics and mechanical properties by varying parameters such as ‐ene structure, light intensity, concentration, and polymer functionality. In contrast to PI‐based photoresins, the absence of ROS in RF photocrosslinking did not affect the expression profile of the tested gene set and resulted in excellent cell viability. Finally, we showed bioprinting proof‐of‐principle with high resolution 2P‐SL using remarkably low RF photoresin concentration (<1.5%), a pivotal aspect in biological applications for enhanced nutrient diffusion and cell migration.^[^ ^40^, ^41^, ^42^ ^]^

## 2 Results and Discussion

### 2.1 Design, Synthesis, and Characterization of Radical‐Free Crosslinker

To prove the feasibility of the proposed approach, we designed a RF photocrosslinker to be hydrophilic, biocompatible, suitable for one and two‐photon applications, and applicable to any photoresin formulation with Michael acceptor (‐ene) moieties. As a starting material we chose PEG4SH (10 kDa), a widely used biocompatible material in the field, commercially available, easy to chemically modify and with 4 reactive groups per molecule, which helps to accelerate the gelation kinetics.^[^ ^43^ ^]^ As PC, we synthesized, as previously reported, the non‐fouling hydrophilic 7‐di‐(carboxymethyl)amino 4‐hydroxymethylcoumarin (PC).^[^ ^34^ ^]^ Coumarins have been extensively used in the past decade and preferred over nitro‐benzyl groups because of their higher one and two‐photon cross section and nontoxic photolysis byproducts.^[^ ^3^ ^]^ The absorption spectrum of the PC extends in the UV–vis range, allowing uncaging at 365–405 nm, wavelengths of choice for the vast majority of current light‐based printers (see Figure S1, Supporting Information). On the other hand, its high two‐photon absorption makes the PC suitable also for two‐photon stereolithography (2P‐SL).

In this study, the PC was coupled to the terminal thiols of PEG4SH via the formation of thiocarbonate bonds (see Figure S2, Supporting Information), resulting in the photocaged RF crosslinker PEG4SPC (Figure 1B). Upon light absorption, a heterolytic bond cleavage results in an intermediate coumarinylmethyl cation, which eventually re‐forms the PC by reacting with H_2_O, and a thiocarbonic acid that then undergoes decarboxylation to give free thiol (PEG4SH) (Figure S3, Supporting Information).^[^ ^26^ ^]^

When caged, thiol groups are not free to react with the ‐ene counterpart. In addition, the PC protects thiols from oxidation that would lead to formation of disulfide bonds and limit the storage potential. The storage stability of PEG4SPC was evaluated with ^1^H‐NMR under two conditions: Dry state (lyophilized) and wet state (solubilized in D_2_O). The purified, lyophilized product was stored at −20 °C and showed no detectable signs of hydrolysis for up to 6 months (Figure 1C). Such a shelf life supports the potential marketability of this product, which would make RF accessible to non‐experts in organic synthesis. Hydrolytic stability was also proven to be ideal when PEG4SPC was kept in solution at room temperature for up to 1 month (Figure 1C). Good stability under wet conditions facilitates daily lab work, overcoming the need to continuously prepare fresh solutions.

### 2.2 Radical‐Free Photocrosslinking

**Figure 2:**
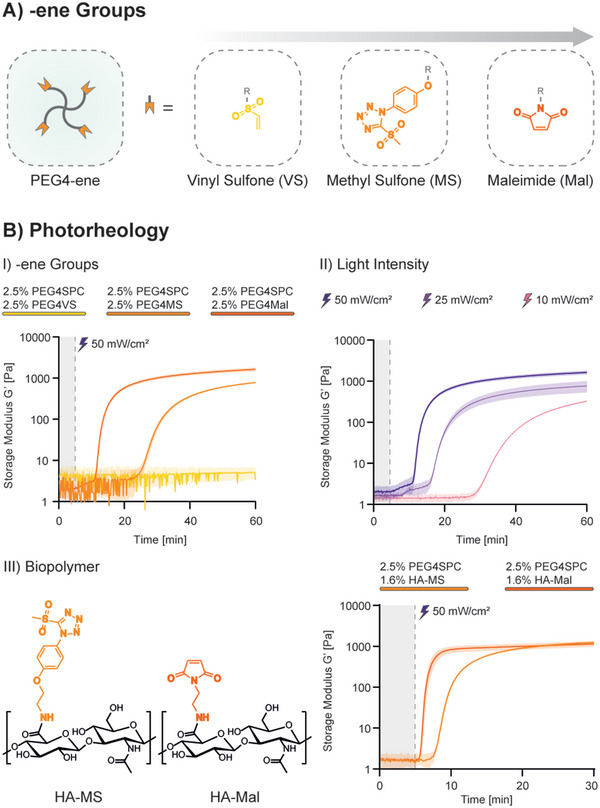
Photorheology analysis of RF crosslinking. A) ‐ene functionalities adopted in this study in ascending order (from left to right) of reactivity. B) I) Photorheology analysis of the various functionalized PEG4‐ene derivatives with RF crosslinker PEG4SPC. After 5 min of measurement, the samples were continuously irradiated with 50 mW cm^−2^ light at 405 nm. As expected, photoresin containing PEG4Mal showed the fastest gelation kinetics, followed by PEG4MS. PEG4VS did not show signs of gelation in the time of the experiment (60 min irradiation). II) Tunable gelation shown with varying light intensity using 2.5% PEG4SPC/2.5% PEG4Mal. III) Chemical structure of HA‐MS and HA‐Mal (left) and photorheology (right) showing improved performances with the use of high molecular weight HA bearing ‐ene groups.

The Michael addition is a robust click reaction that progresses under mild, aqueous conditions.^[^ ^27^ ^]^ However, its kinetics can vary widely, depending on various parameters, such as nature of the ‐ene functionality and pH. To exclude a source of variability, and envisioning biological applications, all the experiments in this study were conducted at physiological pH of 7.4. To study the crosslinking behavior of the developed PEG4SPC, three different 4‐arm‐PEG‐ene counterparts were used: Vinyl sulfone (PEG4VS), aryl‐methyl sulfone (PEG4MS) and maleimide (PEG4Mal) (**Figure** **2**A). While VS and Mal undergo traditional Michael addition, the reaction involving MS is instead a nucleophilic aromatic substitution that yields aryl‐thioether adducts and release of a methylsulfonate anion.^[^ ^44^, ^45^ ^]^ For both reaction mechanisms, the more electron‐deficient the ‐ene is, the more susceptible it is to forming a thioether bond. The order of reactivity is therefore expected to be: PEG4Mal > PEG4MS > PEG4VS. However, on the one hand, vinyl sulfones have slow reaction kinetics, but the thioether adduct that is formed has strong stability. On the other hand, maleimides reacts fast, but the formed thioether is prone to hydrolysis.^[^ ^44^, ^46^, ^47^ ^]^ The aryl‐methyl sulfone selected for this study was only very recently introduced by Paez et al.^[^ ^45^ ^]^ and represents an intermediate condition between maleimides and vinyl sulfones, showing a relatively fast reaction with thiols and good hydrolytic stability.

Photorheology analysis using 405 nm irradiation was performed with 1:1 SH:ene ratio to compare the reactivity of the various photoresin formulations (Figure 2). As expected, PEG4Mal showed the fastest kinetics. The use of PEG4MS resulted in a relevant delay (≈10 min) in gelation, while the photoresin prepared with PEG4VS did not show any sign of gelation after 1 h of light exposure (Figure 2B‐I). By evaluating the uncaging profile of PEG4SPC under the same conditions used for photorheology, the gelation of PEG4Mal/PEG4SPC photoresin was shown to be the only one close to theoretical approximation of Flory‐Stockmayer theory (see Figure S4, Supporting Information). Although our system cannot be considered ideal for various reasons (i.e., possible formation of loops and entanglements, unidirectional light exposure, two‐step reaction process), Flory‐Stockmayer theory approximates the gelation threshold at around 33% (of total bond formation) for an ideal system composed of two tetrafunctional polymers of the same molecular weight and mixed in 1:1 molar ratio. Under the conditions tested, 33% of uncaging was reached after ≈5 min of light exposure, with PEG4Mal gelation starting within the following 5 min.

The significantly lower reactivity of the photoresin containing PEG4MS was first hypothesized to be due to photolysis of the tetrazole ring.^[^ ^48^, ^49^ ^]^ This phenomenon has been commonly reported for irradiation with <300 nm light. NMR analysis revealed perfect stability with 405 nm irradiation under the conditions used in this work (see Figure S5, Supporting Information). Slow crosslinking of PEG4MS formulation can therefore simply be attributed to the slower mechanism of reaction of MS aromatic substitution compared to Michael‐addition using Mal. In addition to varying the ‐ene functionality, gelation tunability was demonstrated by exposing the photoresin to different light intensities (Figure 2B‐II). As expected, the reduction of the light intensity to 25 and 10 mW cm^−2^ produced a slower uncaging (Figure S4, Supporting Information) and a consequently slower crosslinking profile. Interestingly, the most relevant difference in uncaging efficiency between the three intensity conditions tested was found to be within the first 10 min of irradiation. On the other hand, the three conditions reached a similar plateau value of ≈60% uncaged PCs after 1 h.

Although RF photocrosslinking was proven to be possible, light‐based bioprinting methods require much faster gelation kinetics. Following again the Flory‐Stockmayer theory of gelation process, an increase in polymer functionality (*f*) was expected to improve the kinetics of network formation. High molecular weight (≈1.5 MDa) hyaluronic acid (HA) was modified with Mal and, for the first time, with MS (Figure 2B‐III). VS condition was discarded due to the slow kinetics shown with PEG‐based formulations (Figure 2B‐I). Compared to PEG4Mal/MS (*f* = 4), the functionality of the obtained hyaluronic acid methyl sulfone (HA‐MS) (DS: 22%) and hyaluronic acid maleimide (HA‐Mal) (DS: 27.5%) was orders of magnitude higher (f ≈ 800). By combining PEG4SPC with ‐ene functionalized high molecular weight HA, the gel point was estimated to be reached with only 2% of formed thioether bonds. Indeed, the use of HA‐Mal and HA‐MS resulted in a tremendous increase in the speed of RF photocrosslinking (Figure 2B‐III). In particular, HA‐Mal appeared to be the best candidate and was therefore explored for light‐based biofabrication applications.

It is noteworthy to mention that, although photorheology gave an indication of crosslinking kinetics and allowed comparison and selection of photoresin formulations, the presence of a relevant amount of PC, unidirectional irradiation and defined gap distance can lead to underestimation/overestimation of the photoresin performance. The PC used in this work has, at 405 nm, an extinction coefficient 100 times higher than LAP (Figure S1, Supporting Information). Therefore, the higher the concentration of PEG4SPC, the stronger the absorption of the photoresin. As an example, if a 2.5% PEG4SPC were to be used, Beer‐Lambert law estimates a total light absorption with a path length of only ≈260 µm. A simple variation in PEG4SPC concentration or gap distance could therefore significantly impact the rheological output (see Figure S6, Supporting Information). Due to the inherent high absorption of RF photoresins, this chemical strategy does not appear suitable for technologies in which light penetration is fundamental, like VP.^[^ ^10^, ^11^, ^50^ ^]^ On the other hand, RF appears suitable for layer‐by‐layer stereolithography where light absorption is a desirable property and commonly sought through the addition of photoabsorbers.^[^ ^5^, ^51^, ^52^, ^53^, ^54^ ^]^

### 2.3 Cellular Response to Absence of Reactive Oxygen Species

**Figure 3:**
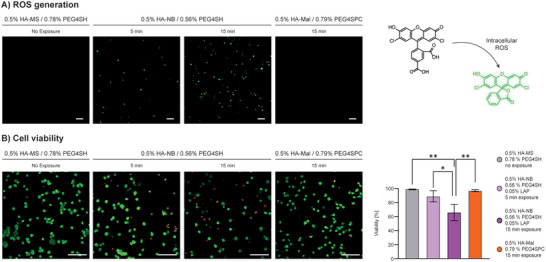
A) ROS fluorescence assay of cell‐laden HA‐Mal/PEG4SPC and HA‐NB/PEG4SH/LAP hydrogels formed under 405 nm irradiation (15 min, 50 mW cm^−2^). HA‐MS/PEG4SH cell‐laden hydrogel was also included as non‐photocaged, non‐irradiated control. On the right, illustration of intracellular ROS driven oxidation of non‐fluorescent compound (black) to highly fluorescent 2’,7’‐dichlorofluorescein (green). While for LAP containing photoresin the cellular response to ROS was evidenced by green fluorescence, RF photoresin showed no detectable signal as for the non‐irradiated HA‐MS/PEG4SH control. Moreover, for LAP‐containing photoresins, an increase in light exposure (15 min vs 5 min) resulted in an increase in ROS positive (green) cells. Scale bar: 100 µm. B) Live/Dead assay of cell‐laden hydrogels (405 nm irradiation, 5 or 15 min, 50 mW cm^−2^) showed excellent viability for RF system and significant cell death for free‐radical based photoresin after 15 min of irradiation that can be attributed to ROS generation. Scale bar: 100 µm.

Design, synthesis and photochemical characterization of RF photoresins proved the core hypothesis of this work to be feasible. However, to translate such a chemical strategy to biological applications, it must prove to be biocompatible. First, the difference between free‐radical (LAP‐based) and RF (PC‐based) systems in terms of radical generation and deleterious impact on cell viability was assessed with ROS and Live/Dead assays using cell‐laden photoresins (**Figure** **3**). As a free‐radical comparison to the selected HA‐Mal‐based formulation, we chose HA‐norbornene (HA‐NB), which undergoes fast radical‐initiated photoclick step‐growth reactions with a thiolated crosslinker. To better compare the two formulations, HA‐NB was synthesized to have a similar degree of substitution (DS: ≈18%) to that of HA‐Mal. Instead of PEG4SPC, HA‐NB was mixed with non‐caged PEG4SH and 0.05% LAP. As a non‐photocaged, non‐irradiated control we chose HA‐MS/PEG4SH which, in contrast to the essentially instantaneous crosslinking of HA‐Mal/PEG4SH, allows one to mix the precursor solutions in the presence of cells and form a stable hydrogel in few minutes (Figure S7, Supporting Information). In addition, while the optimized RF photoresin requires similar irradiation time (15 min) when compared to widely used chain‐growth crosslinking strategies (i.e., HA‐methacryloyl, see Figure S7, Supporting Information), high performance photoresins such as HA‐NB/PEG4SH can require much less (5 min). The resulting hydrogels embedded with primary normal human dermal fibroblasts (NHDF) showed significant differences both in terms of ROS detection and cell viability. The intracellular ROS‐triggered oxidation of the fluorogenic dye 2’,7’‐dichlorofluorescin diacetate (DCFDA) was clearly detectable (green fluorescence) for the cells in HA‐NB/PEG4SH hydrogel exposed for 5 or 15 min. In contrast, no ROS‐positive cells were found in the RF HA‐Mal/PEG4SPC gels and in the unexposed HA‐MS/PEG4SH control (Figure 3A). Excellent cell viability (≈100%) was reported for RF photoresin upon gelation (day 0), while the impact of ROS in free radical‐based photoresin resulted in considerable cell death with increasing irradiation time (Figure 3B).

**Figure 4:**
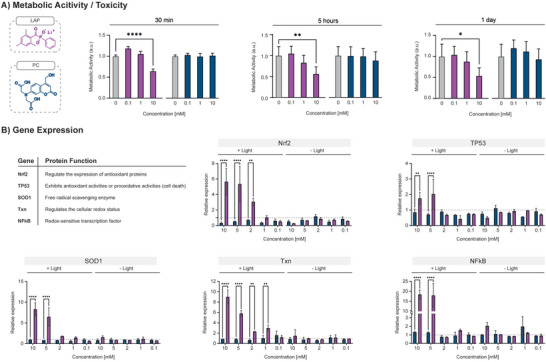
Comparison of LAP and PC for biological applications. A) MTS assay on NHDF incubated with LAP (pink) or PC (blue) at different concentrations for 30 min, 5 h, and 1 day. Cells showed a significant decrease in metabolic activity only when LAP was used at 10 mm. The PC used in this study did not show any deleterious effect on NHDF under the conditions tested. B) Gene expression analysis targeting ROS responsive genes for NHDF exposed to various concentrations of LAP and PC. Samples were irradiated with 405 nm light (6.8 mW cm^−2^) for 15 min and the results compared to non‐irradiated samples. As expected, non‐irradiated samples showed no up‐regulation of target genes. In the absence of light, no radicals are, in fact, produced by the compounds under study. In the presence of light, samples exposed to LAP showed pronounced upregulation of genes involved in antioxidant and radical scavenging activities. A negligible effect was only reported for the lowest concentration (0.1 mm). Remarkably, for samples incubated with PC, upregulation was not reported under any condition.

The coumarin uncaging process is known to not generate harmful radicals, as also proved in **Figure** **4**, but for the system to be fully biocompatible, the PC itself needs to show no signs of cytotoxicity. For example, the photolysis byproducts of nitrobenzyl derivatives (a commonly used class of photoremovable groups in tissue engineering) are reactive and potentially toxic aldehydes and ketones.^[^ ^3^, ^26^ ^]^ In the case of the PC used in this work, the uncaging reaction simply releases 7‐di‐(carboxymethyl)amino 4‐hydroxymethylcoumarin (PC) and a CO_2_ molecule (Figure S3, Supporting Information). To assess whether this molecule has a deleterious effect on human cells, we performed an MTS assay (Figure 4A). The impact of PC on NHDFs was compared to LAP, the state‐of‐the‐art PI for light‐based printing. Various incubation times (30 min, 5 h, and 1 day) and concentrations (0.1, 1, and 10 mm) were tested. The assay showed a significant reduction in metabolic activity only for LAP and when used at highest concentration. PC was shown to have no toxic effect on cells with incubations up to 1 day, suggesting high biocompatibility of the molecule even in cases of a long printing process.

A more in‐depth analysis on the impact of PC versus LAP in the presence or absence of light was conducted with gene expression analysis, focusing on the cellular response to ROS. We have chosen, based on the literature, SOD1, Nrf2, NF‐*κ*B, Txn, and tumor suppressor protein 53 (TP53) genes to track the multifaceted response to ROS from different perspectives. Superoxide dismutase (SOD) overexpression is a consequence of ROS presence in the cellular environment.^[^ ^55^, ^56^, ^57^, ^58^ ^]^ SOD catalyzes conversion of superoxide to oxygen and hydrogen peroxide, acting as a first‐line defense against the potential toxicity of these molecules.^[^ ^59^ ^]^ Nrf2 and NF‐*κ*B are transcription factors whose activation by elevated ROS enables the expression of antioxidant pathways as thioredoxin (Txn),^[^ ^60^ ^]^ and detoxification enzymes as glutathione S‐transferases.^[^ ^61^, ^62^ ^]^ TP53 is a transcription factor linked to DNA damage resulting from different stimuli, with the role of avoiding propagation of mutated genomes by inducing cell cycle arrest, senescence or apoptosis.^[^ ^63^ ^]^ Overall, an upregulation of these genes is a consequence of an ROS accumulation in the cellular environment which requires a fast response to avoid toxicity and mutagenesis.

Of particular interest for the bioprinting community, the alteration of the cellular redox state, was found to induce cell differentiation and impact cell proliferative capacity.^[^ ^64^ ^]^ A cell can thus remain viable thanks to the activation of the above‐mentioned protective pathways, but might hide some significant changes. When printing is performed with delicate primary or stem cells, highly susceptible to alteration of intracellular and extracellular environment, the removal of a source of variability (ROS via RF strategy) is desirable.

When kept in the dark, primary NHDFs did not show signs of upregulation of the tested genes, in accordance with the hypothesis that ROS are not generated when the photosensitive molecules (LAP or PC) are not excited (Figure 4B). In contrast, light exposure triggered a significant upregulation of the investigated genes in the presence of LAP. Cells therefore sensed and responded to the increase of ROS generated with LAP excitation. Notably, no gene upregulation was detected for NHDF incubated with PC. The striking difference between the two conditions revealed substantially unexplored territory in in this field, suggesting the need for a more in‐depth understanding of the cellular response to light‐based methods.

### 2.4 Two‐Photon Stereolithography (Bio)Printing

**Figure 5:**
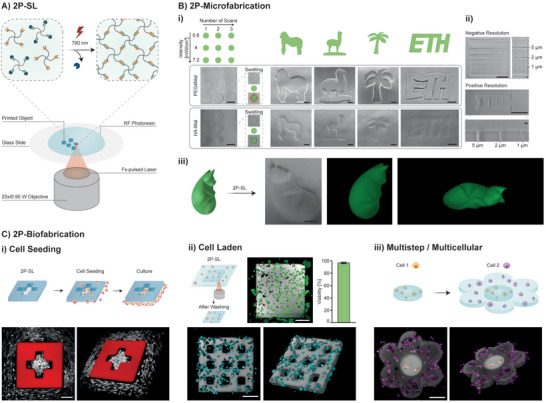
2P‐SL RF (bio)printing. A) Illustration of 2P‐SL with RF photoresin. B) i) Printing parameter screening and examples of printed designs. The use of HA‐Mal is shown to drastically reduce the swelling (orange) and therefore better reproduce the desired shapes (green). Scale bars: 100 µm. ii) Negative (writing) and positive (line width) resolution for the optimized HA‐Mal‐based photoresin. Scale bars: 100 µm and 5 µm in close ups. iii) Example of 2P‐SL of a 3D object. A .stl model of a “sleeping cat” was printed using HA‐Mal/PEG4SPC photoresin and imaged exploiting residual PC fluorescence. Scale bar: 100 µm. C) Proof‐of‐concept of 2P‐based biofabrication with optimized HA‐Mal/PEG4SPC photoresin. i) Illustration of fabrication and post‐seeding procedure (top). 3D “Swiss flag”‐like microwell (red) and post seeded NHDF (white) (bottom). ii) 2P‐SL in the presence of cells (NHDF) showed excellent viability upon printing (day 0) (top). Example of bioprinting of user‐defined shape (grey) in the presence of cells (blue) (bottom). iii) Illustration of multistep/multicellular printing concept (top) and actual bioprinted multicellular object (bottom). Scale bars: 100 µm.

Overall, RF photocrosslinking was proven to be effective, tunable and highly biocompatible. In this final section, proof‐of‐concept of RF applicability for light‐based printing technologies is presented using 2P‐SL (**Figure** **5**A). This method is based on the scanning of a tightly focused femtosecond‐pulsed infrared laser and offers the highest printing resolution in the field.^[^ ^3^, ^8^, ^65^ ^]^ It requires fast reaction kinetics and has been traditionally used with highly functionalized polymers and high photoresin concentrations (i.e., 10–15% Gel‐MA or Gel‐NB with DS: ≈90%).^[^ ^66^, ^67^ ^]^

Thanks to the significant two‐photon absorption cross section of coumarin derivatives, this class of photoremovable groups has been used for applications involving 2P uncaging processes.^[^ ^33^, ^68^, ^69^ ^]^ The PC used in this work was therefore hypothesized to also be suitable for 2P‐SL (bio)printing.

First, a parameter screening was performed (Figure 5B). The laser intensity and the number of scans were varied to identify the optimal printing conditions. Cylinders of 70 µm diameter and height were designed using the region‐of‐interest (ROI) function, which allows the user to demarcate the areas to be scanned. Upon washing of uncrosslinked resin, cylindrical objects were observable by phase‐contrast imaging. In accordance with photorheological analysis (Figure 2, Figure S4, Supporting Information), a low light intensity or light dose (number of scans) can lead to insufficient uncaging and no gelation (i.e., no cylinder formation) or low uncaging, thus leading to soft gels with lower crosslinking density (higher swelling degree). Notably, the PEG4Mal‐based formulation resulted in significant swelling. Again, HA‐Mal‐based formulation performed better, as no swelling was observed. This different degree of swelling can be attributed to a combined effect of reaction kinetics and initial concentration of photocage that, upon removal, is washed out leading to favorable water uptake (Figure S8, Supporting Information). The swelling behavior can strongly affect the final printing fidelity. As an example, ROI designs depicting a gorilla, alpaca, palm tree, and the ETH logo were printed using the optimal laser dose, which was identified using the screened values (Figure 5B). While structures printed with PEG4Mal/PEG4SPC swelled significantly, deviating from the original dimensions, HA‐Mal/PEG4SPC printed objects replicated the desired shapes with high fidelity. To better evaluate the capabilities of this RF photoresin, maximum resolution was investigated. Writing resolution (or negative resolution) is defined as the minimum distance between two printed objects and was found to be ≈1 µm (Figure 5B‐ii). Line width resolution (or positive resolution), which refers to the minimum feature size that can be printed, was found to be ≈1 µm. Considering the objective set‐up (25×, 0.95 NA, water immersion), both negative and positive resolutions came close to matching the theoretical maximum, proving excellent performance of the optimized RF system. Optimal photoresin and laser parameters were used to print the 3D model of a “sleeping cat.” By using a script previously developed in our lab,^[^ ^34^ ^]^ which converts an .stl model into a stack of ROIs, it was possible to use a commercial 2P‐microscope (Leica SP8 equipped with Spectra Physics Mai Tai laser) as a 2P‐SL printer. Upon washing of uncrosslinked resin and profiting from the fluorescence of residual PC molecules, 3D reconstruction of the printed model was possible via confocal imaging (Figure 5B‐iii).

Building on the findings described above, RF 2P‐SL bioprinting was explored (Figure 5C). First, a “Swiss flag”‐shaped microwell was printed and post‐seeded with NHDF (Figure 5C‐i). Consistent to what has been reported for one‐photon crosslinking (Figure 4), 2P‐SL in the presence of cells was also shown to be highly biocompatible (Figure 5C‐ii). Excellent viability upon printing (≈100%) also indicated that no photodamage due to the 2P laser scanning occurred under optimized conditions (7.2 mW cm^−2^, 3 scans). A cell‐laden grid was generated to highlight the possibility of bioprinting diverse shapes (Figure 5C‐ii, bottom). Finally, a multistep approach was adopted to bioprint a multicellular flower‐shaped construct (Figure 5C‐iii). A first printing step used NHDFs pre‐incubated with a first fluorescent cell tracker. After washing of uncrosslinked resin, a second printing step used photoresin with NHDFs incubated with a second photo‐orthogonal cell tracker. As a result, a multicolor/multicellular flower‐like print was obtained.

Notably, all the 2P‐SL printing was performed with a remarkably low polymer concentration (0.5% HA‐Mal) compared to the standards of this method. This highlights the excellent kinetics of the presented RF process and its potential suitability for a variety of printing methods relying on fast gelation. The use of low polymer concentration is of particular interest for tissue engineering applications, since the resulting hydrogels have generally better properties for cell migration, spreading and matrix deposition (i.e., larger mesh size, better diffusivity of nutrients, softness).^[^ ^40^, ^41^, ^42^ ^]^ For example, as HA‐based soft matrices are considered good candidates for brain‐mimicking artificial ECM, neuronal cells were bioprinted showing formation of neurite extensions (Figure S9, Supporting Information).^[^ ^70^, ^71^ ^]^

## 3 Conclusion

In summary, a radical‐free (RF) photocrosslinking approach was demonstrated. It offers a valid alternative to common free‐radical strategies that use potentially toxic PIs and radical‐initiating species. RF photocrosslinking was demonstrated to be fast, tunable and suitable to one‐ and two‐photon excitations. High biocompatibility and negligible ROS generation were also established, making this approach a potentially beneficial tool for bioprinting in the presence of radical‐sensitive molecules and cells (i.e., cell/drug delivery with 3D printed microcarriers/microrobots). In addition, the possibility of synthesizing RF crosslinker in gram scale and storing it with high stability makes RF a potentially marketable off‐the‐shelf solution. We believe that this seminal work will also stimulate the generation of a variety of RF crosslinkers having different polymer backbones, functionality, and also different PCs, opening to potentially orthogonal uncaging wavelengths. In particular, we anticipate that a significant improvement will come with the introduction of a PC that upon uncaging undergoes a shift or a strong reduction of the absorption spectrum, so that cleaved molecules do not limit the uncaging kinetic and thus the crosslinking reaction. In addition, to strengthen its potential for one‐photon bioprinting and in vivo biomedical applications, the RF crosslinking will benefit from the use of near‐IR cleavable PCs that would provide improved tissue penetration. Beside the clear potential in one‐ and two‐photon stereolithography, with the above‐mentioned advances that would further improve the reaction kinetics and penetration depth, RF crosslinking could also find use in extrusion 3D bioprinting where relatively thick filaments (≈400 µm) are commonly extruded in the presence of cells.

## 4 Experimental Section

All chemicals were purchased from Merck and cell culture reagents from Gibco unless indicated otherwise. All cell experiments were performed using NHDFs isolated from juvenile foreskin skin biopsies. Biopsies were taken under parental informed consent and their use for research purposes was approved by the Ethical Committee of Canton Zurich (BASEC‐Request‐Nr. 2018‐00269).

### Synthesis and Characterization of Photocage (DCMAC‐OH and PC)

7‐di‐((*tert*‐butyl‐carboxy)methyl)amino 4‐hydroxymethylcoumarin (DCMAC‐OH) was synthesized in gram scale as previously described.^[^ ^34^ ^]^ The compound was purified by C18 preparative HPLC (Agilent) in a gradient from 10% to 100% ACN in H_2_O with 0.1% TFA. Identity of the compound was verified by ^1^H‐NMR (Bruker Ascend 500 MHz, 128 scans, see Figure S10, Supporting Information) and LC‐MS (m/z: [M + H] calcd 419.19, found, 420). *Tert*‐butyl deprotection was performed with acid treatment in 1:1 DCM:TFA for 15–30 min. After removal of DCM and TFA by evaporation under nitrogen stream, the product 7‐(dicarboxymethylamino)‐4‐(hydroxymethyl)‐coumarin (PC) was purified by C18 preparative HPLC (Agilent) in a gradient from 10% to 100% ACN in H_2_O with 0.1% TFA. Identity of the compound was verified by LC‐MS (m/z: [M + H] calcd 307.07, found, 307). For analysis of absorption spectrum and extinction molar coefficient at 405 nm (*ε*_405_), PC was dissolved in HEPES 25 mm, 150 mm NaCl pH 7.4 at 10 mm final concentration. Micro‐volume UV–vis absorbance analysis were performed at 25 °C, from 300 to 700 nm with 1 nm step (Synergy H1, BioTek). The same procedure was used for LAP.

### Synthesis of 7‐di‐((*tert*‐butyl‐carboxy)methyl)amino coumarin 4‐yl)methyl carbonate (DCMAC‐NPC)

DCMAC‐NPC synthesis was adapted from a previously published protocol.^[^ ^34^ ^]^ 100 mg (0.24 mmol) of DCMAC‐OH were dissolved in 1 mL of dry DCM with 50 µL *N*,*N*‐diisopropylethylamine (DIPEA). 48 mg (0.24 mmol) of 4‐nitrophenyl chloroformate (NPC) pre‐dissolved in 1 mL of DCM were added dropwise to this solution while stirring. The reaction, monitored by HPLC (Hitachi), appeared to be completed in around 20 min. The reaction mixture was dried under reduced pressure and used for the synthesis of PEG4SPC without further purification.

### Synthesis and Stability Tests of Photocaged 4‐arm‐peg‐thiol (PEG4SPC)

650 mg of 10 kDa PEG4SH (JenKem Technology USA) (0.26 mmol of SH, 1 eq.) were dissolved in 2 mL of dry DMF in the presence of 475 µL DIPEA (2.7 mmol, 10 eq.). 227 mg of DCMAC‐NPC (0.39 mmol, 1.5 eq.) were dissolved in 1 mL of dry DMF and added dropwise to the solution under stirring. Reaction was monitored with HPLC (Hitachi) and found to be completed after 30 min. *Tert*‐butyl deprotection was performed with acid treatment in by adding TFA to a total 1:5 DMF:TFA ratio for 15–30 min. After deprotection, TFA was removed by evaporation under nitrogen stream. PEG4SPC was further diluted in 10 mL of ACN and purified by C18 preparative HPLC (Agilent) in a gradient from 10% to 90% ACN in H_2_O with 0.1% TFA. Identity of the compound was verified by ^1^H‐NMR (see Figure S11, Supporting Information).

Stability of the RF crosslinker PEG4SPC was determined with ^1^H‐NMR (Bruker Ultrashield 400 MHz, 1024 scans) by evaluating the ratio of the chain‐end methylene protons close to caged thiols (t, 2H, CH_2_, 3.16 ppm) and close to uncaged thiols (t, 2H, CH_2_, 2.75 ppm) (see Figure 1C and Figure S11, Supporting Information). Lyophilized product stored at −20 °C was dissolved in D_2_O and analyzed right after freeze‐drying and after 6 months of storage. For the stability of PEG4SPC in solution, the product was dissolved in D_2_O, kept at room temperature (protected from light) and analyzed after 4 weeks.

### Synthesis of 2‐(4‐(5‐(methylsulfonyl)‐1H‐tetrazol‐1‐yl)phenoxy)ethan‐1‐amine (MS)

The synthesis was performed as recently reported by Paez et al.^[^ ^45^ ^]^ The final product (MS) was purified by C18 preparative HPLC (Agilent) in a gradient from 10% to 90% ACN in H_2_O with 0.1% TFA. Identity of the compound was verified by ^1^H‐NMR in DMSO‐d6 (see Figure S12, Supporting Information) and LC‐MS (m/z: [M + H] calcd 283.07, found, 284). Photostability of MS was investigated by irradiating a 2.5% solution of the product in D_2_O under the conditions used for photorheology (405 nm, 200 µm gap, 50 mW cm^−2^) for 30 min and then analyzed with ^1^H‐NMR (see Figure S5, Supporting Information)

### Synthesis of 4‐arm‐PEG‐methyl sulfone (PEG4MS)

The synthesis was performed as recently reported by Paez et al.,^[^ ^45^ ^]^ using 10k Da PEG4‐succinimidyl carboxymethyl ester (PEG4SCM, Creative PEGWorks). The product was purified by C18 preparative HPLC (Agilent) in a gradient from 10% to 90% ACN in H_2_O with 0.1% TFA. Identity of the compound was verified by ^1^H‐NMR in D_2_O (Bruker Ascend 500 MHz, 128 scans, see Figure S13, Supporting Information).

### Synthesis of Hyaluronic Acid Methyl Sulfone

125 mg (0.3 mmol, 1 eq.) of high molecular weight HA (1.5 MDa, HTL Biotechnology) were left to dissolve overnight, under stirring, in 50 mL 150 mm MES buffer pH 4.5. Then, 71.25 mg of 1‐Ethyl‐3‐(3‐dimethylaminopropyl)carbodiimide (EDC, 3.7 mmol, 1.2 eq.) and 43.15 mg (1.2 eq.) of N‐hydroxysuccinimide (NHS) were dissolved in 5 mL of 150 mm MES buffer pH 4.5 and quickly transferred to the reaction mixture. Finally, 106 mg of MS (1.2 eq.) previously dissolved in 1 mL H_2_O were added dropwise. The reaction was left to proceed under stirring for 24 h, dialyzed against acidified mQ H2O (pH 4 using HCl) for 5 days with frequent water changes and freeze‐dried. The degree of substitution (DS) of the resulting HA‐MS was estimated with ^1^H‐NMR in D_2_O using internal standard 3‐(trimethylsilyl)‐1‐propanesulfonic acid (DSS, 2H, ≈0.75 ppm) and MS aromatic ring protons (≈7.4 and 7.8 ppm, see Figure S14, Supporting Information).

### Synthesis of Hyaluronic Acid Maleimide

125 mg (0.3 mmol, 1 eq.) of high molecular weight HA (1.5 MDa, HTL Biotechnology) were left to dissolve overnight, under stirring, in 50 mL 150 mm MES buffer pH 4.5. Then, 71.25 mg of 1‐ethyl‐3‐(3‐dimethylaminopropyl)carbodiimide (EDC, 3.7 mmol, 1.2 eq.) and 43.15 mg (1.2 eq.) of *N*‐hydroxysuccinimide (NHS) were dissolved in 5 mL of 150 mm MES buffer pH 4.5 and quickly transferred to the reaction mixture. Finally, 91 mg of *N*‐(2‐aminoethyl)maleimide trifluoroacetate salt (1.2 eq.) previously dissolved in 1 mL H_2_O were added dropwise. The reaction was left to proceed under stirring for 24 h, dialyzed against acidified mQ H2O (pH 4 using HCl) for 5 days with frequent water changes, and freeze‐dried. The degree of substitution (DS) of the resulting HA‐Mal was estimated with ^1^H‐NMR in D_2_O using *N*‐acetyl protons peak (≈2 ppm) and maleimide double bond peak (≈7 ppm, see Figure S15, Supporting Information).

### Synthesis of Hyaluronic Acid Norbornene

2.5 g (6.25 mmol, 1 eq.) of high molecular weight HA (1.5 MDa, HTL Biotechnology) were left to dissolve overnight, under stirring, in 1 L of 150 mm MES buffer pH 4.5. Then 5.45 g of adipic acid dihydrazide (ADH, 31.25 mmol, 5 eq.) were added to the solution. When completely dissolved, 300 mg of 1‐ethyl‐3‐(3‐dimethylaminopropyl)carbodiimide (EDC, 1.56 mmol, 0.25 eq.) were solubilized in 2 mL of 150 mm MES buffer pH 4.5 and added dropwise to the reaction mixture. After 4 h the pH was found to be 4.72 and the reaction was quenched by addition of NaOH 1 m to reach pH 7. After addition of 2 g of NaCl the solution was dialyzed against mQ H_2_O for 5 days with frequent water changes and freeze‐dried. After lyophilization, 2.5 g of HA‐ADH were left to dissolve overnight, under stirring, in 1 L of PBS pH 7.4. Then, 2 g of carbic anhydride (12.5 mmol, 2 eq.) were dissolved in 10 mL of DMF and added dropwise to the solution. The reaction was left to proceed for 8 h with pH adjustments every 30 min using NaOH 2 m to maintain pH 7.4. After addition of 2 g of NaCl the solution was dialyzed against mQ H_2_O for 3 days using a tangential flow filtration system (ÄKTA Flux, Cytiva) and then freeze‐dried. HA‐NB degree of substitution (DS) was found to be 18% with ^1^H‐NMR (Bruker Ultrashield 400 MHz, 1024 scans) integrating norbornene double bond peak (≈6.3 ppm, see Figure S16, Supporting Information). For NMR analysis the high viscous polymer was solubilized at 5 mg mL^−1^ in 1 mL of 2 mm NaCl D_2_O solution in the presence of a known amount of internal standard 3‐(trimethylsilyl)‐1‐propanesulfonic acid (DSS). High ionic strength was found to be a powerful tool to improve the quality of the spectra. As reported by Ret et al., for long‐chain, high viscous polymers in particular, the control of their conformation in solution can determine better proton mobility.^[^ ^72^ ^]^

### MTS Assay

NHDFs cultured in DMEM + GlutaMAX‐I + 10% fetal bovine serum + 10 µg mL^−1^ gentamicin were seeded at passage 7 in 96 well plate at 50 000 cells per well. Cells were left to adhere overnight, and then the medium was changed to serum‐free 24 h before treatment. Cells were then cultured in medium containing LAP (0.1, 1, 10 mm), PC (0.1, 1, 10 mm) or control medium (no LAP, no PC) for 30 min, 5 h, and 1 day. Each condition was tested in triplicate (*n* = 3). Following provider (abcam) protocol, media was changed with fresh serum‐free media containing 20 uL of MTS reagent. Color was left to develop for 3.5 h under standard culture conditions prior to measuring absorbance at 490 nm with a microplate reader (Synergy H1, BioTek).

### Gene Expression Analysis

Gene expression levels were assessed through real‐time PCR (qPCR) analysis. NHDF (passage 8) were plated and grown to confluence. Medium was changed to serum‐free for 24 h before treatment. Cells were incubated with medium containing PC or LAP at different concentrations (0.1, 1, 2, 5, 10 mm) for 30 min and then exposed to 405 nm light (≈6.8 mW cm^−2^) for 15 min. After light exposure, the media was changed for fresh, serum‐free media without PC or LAP. RNA was extracted after 8 h with NucleoZol (Macherey‐Nagel) following manufacturer's instructions and retrotranscribed to cDNA with GoScript Reverse Transcriptase (Promega). After 1:5 dilution with RNAse‐free water, cDNA was used for qPCR analysis with GoTaq PCR mix (Promega) on a QuantStudio 3 device (Applied Biosystems). Analyzed genes were SOD1 (Fw: CCTAGCGAGTTATGGCGACG, Rv: CCACACCTTCACTGGTCCAT), NF‐*κ*B (Fw: GCTTAGGAGGGAGAGCCCAC, Rv: AACATTTGTTCAGGCCTTCCC), Txn (Fw: CTTGGACGCTGCAGGTGATA, Rv: AGCAACATCATGAAAGAAAGGCT), Nrf2 (Fw: AGGTTGCCCACATTCCCAAA, Rv: AGTGACTGAAACGTAGCCGA), TP53 (Fw: CGCTTCGAGATGTTCCGAGA, Rv: CTTCAGGTGGCTGGAGTGAG). Values were all normalized to GAPDH (Fw: AGTCAGCCGCATCTTCTTTT, Rv: CCAATACGACCAAATCCGTTG). Statistical analysis was performed with Prism using two‐way ANOVA.

### Photorheology

Photorheology analyses were carried out on an Anton Paar MCR 302e equipped with a 10 mm parallel plate geometry and 6 mm glass floor. An Omnicure Series1000 lamp (Lumen Dynamics) was used as a light source. Sequential 400–500 nm and narrow 405 nm bandpass filters (Thorlabs) were placed within the optical fiber path. Light intensity at the sample site was measured with a PM101 power meter (Thorlabs) equipped with a photodiode power sensor (S120VC, Thorlabs). The various photoresin components PEG4SPC, PEG4‐VS/MS/Mal, HA‐Mal/MS/NB and HA‐MA (DS: 20–50%, Mw 50–70 kDa, Sigma) were dissolved in NaCl 150 mM, 25 mm HEPES buffer pH 7.4 at 10% (PEG‐based) or 2% (HA‐based). pH was checked and adjusted if necessary to 7.4 using NaOH 1 m. Photoresins with 1:1 SH:ene molar ratio, and various concentrations were then prepared from this starting stock solution by mixing (pipetting) and diluting in NaCl 150 mm, 25 mm HEPES buffer pH 7.4. All procedures were performed in the dark. All tests were performed at 25 °C in the presence of a wet tissue paper in the chamber to prevent the sample from drying. Oscillatory measurements were performed in triplicate (*n* = 3) using 18 uL photoresins at 2% shear rate and 1 Hz frequency with 200 or 500 µm gap and 10 s measuring point duration.

### Uncaging Test

A solution of 2.5% PEG4SPC in 150 mm NaCl, 25 mm HEPES buffer pH 7.4 was prepared as described above. Using the same photorheology conditions described above (light intensity: 50, 25, and 10 mW cm^−2^, gap distance: 200 µm), but a larger parallel plate (20 mm), 74 µL of the solution were irradiated for 1, 2, 10, 30, and 60 min. 50 µL of this solution were then diluted 1:10 with H_2_O and used for HPLC analysis (Hitachi) in a gradient from 10% to 90% ACN in H_2_O with 0.1% TFA. Uncaging was estimated by tracking the reduction of the PEG4SPC peak on the 385 nm trace compared to the non‐exposed PEG4SPC solution.

### Cell Laden Photocrosslinking and Live/Dead Assay

RF photoresins composed of 0.5% HA‐Mal and 0.79% PEG4SPC were prepared as indicated above by solubilizing the polymers in 150 mm NaCl 25 mm MES buffer pH 7.4. Free‐radical photoresin was prepared with 0.5% HA‐NB, 0.56% PEG4SH and 0.05% LAP in the same buffer. Solutions were filtered sterilized through 0.20 µm filters. Then, NHDFs were resuspended in these photoresins at 1 million cells mL^−1^ and the resulting bioresin was pipetted (15 µL) into PDMS molds (rings with 4 mm internal diameter, 6 mm external diameter, 1 mm height) positioned onto 8‐well glass chambers (Nunc Lab Tek, ThermoScientific). Glass chambers and PDMS molds were previously sterilized via sequential EtOH 70% washing and UV‐treatment. The samples were crosslinked for 15 min using the same photorheology setting described above, with 405 nm light, 50 mW cm^−2^ intensity. Control photoresin composed of 0.5% HA‐MS, 0.78 PEG4SH in 150 mm NaCl 25 mm MES buffer pH 7.4 was left to crosslink for 20 min with no light exposure. After crosslinking, FluoroBrite DMEM supplemented with 1:2000 CalceinAM (Invitrogen), 1:500 Propidium Iodide (PI, Fluka) was added. After 40 min, imaging was performed on a Leica SP8 microscope (Leica) equipped with a 25× objective. Z‐stacks were acquired from the sample surface at 2 µm steps and 100 µm into the sample. The ensuing pictures resulted from maximum intensity z‐projection. Cell viability was assessed by counting viable (CalceinAM) and dead (PI) cells with the ImageJ Analyze particle function.

### Reactive Oxygen Species Assay

Hydrogels were prepared as indicated above, using 2 million cells mL^−1^. The resulting bioresins were pipetted (15 µL) into PDMS molds positioned onto 8‐well glass chambers (Nunc Lab Tek, ThermoScientific). Glass chambers and PDMS molds were previously sterilized via sequential EtOH 70% washing and UV‐treatment. The samples were crosslinked for 15 min using the same photorheology setting described above, with 405 nm light, 50 mW cm^−2^ intensity. After crosslinking, the cellular ROS assay (ab113851, abcam) was performed following provider's instructions. Serum‐free DMEM supplemented with 20 mm DCFDA was added to the wells. After 4 h, imaging was performed on Leica TCS SP8 (Leica) confocal microscope equipped with a 10× objective. Images were taking using the same parameters and elaborated using Fiji, subtracting the same background level (identified with HA‐MS/PEG4SH non‐exposed control).

### Two‐Photon Stereolithography

RF photoresin composed of 0.5% HA‐Mal and 0.79% PEG4SPC was prepared as previously described and pipetted (15 µL) into PDMS molds (ring with 4 mm internal diameter, 6 mm external diameter, 1 mm height) positioned onto 8‐well glass chambers (Nunc Lab Tek, ThermoScientific). 2P‐SL was performed using a Leica TCS SP8 (Leica) confocal microscope equipped with a Mai Tai two‐photon laser (Spectra‐Physics) tuned at 780 nm. ROIs were designed in the desired shape (circle, gorilla, alpaca, palm, ETH logo) using LAS X software functionalities. For parameter screening, a thermal power sensor (S175C, Thorlabs) was used to determine the light intensity at various laser output power. 2P‐SL was performed with HCX IRAPO 25X/0.95NA water immersion objective, 1 µm z‐step, 600 Hz scanning, bi‐directional scanning, 1024 × 1024 format and zoom factor of 1. Imaging was performed on EVOS M5000 imaging system (ThermoFisher) equipped with 4× and 10× objectives.

3D model “Sleeping cat” was printed using a script previously developed in the lab (available at https://github.com/nbroguiere/F2P2). The script slices the .stl model into a sequence of ROIs which are then executed under the LAS X Live Data Mode. To ensure attachment to the glass slide, the printing was set to start ≈5 µm below the glass surface. After extensive washing of uncrosslinked photoresin, imaging of the 3D model was performed on Leica SP8 microscope (Leica) equipped with a 25× objective, taking advantage of two‐photon triggered fluorescence of residual photocage.

### Two‐Photon Biofabrication and Live/Dead Assay

RF photoresin composed of 0.5% HA‐Mal and 0.79% PEG4SPC was prepared as previously indicated. The resulting bioresin was pipetted (15 µL) into PDMS molds (ring with 4 mm internal diameter, 6 mm external diameter, 1 mm height) positioned onto 8‐well glass chambers (Nunc Lab Tek, ThermoScientific). Glass chambers and PDMS molds were previously sterilized via sequential EtOH 70% washing and UV‐treatment. 2P‐SL was performed as previously indicated using ROI functionality. For the “cells seeding” example of Figure 5C‐i, after 2P‐SL the uncrosslinked resin was washed out with 5× media changes. Then, a suspension of NHDF (1 million cells mL^−1^) was added and cells were left to adhere for 1 h. After washing 3× with media, cells were left to spread for 3 days. Then, FluoroBrite DMEM supplemented with 1:2000 CalceinAM (Invitrogen) was added and after 40 min, imaging was performed on a Leica SP8 microscope (Leica) equipped with a 25× objective.

For the cell‐laden applications (Figure 5C‐ii,iii), NHDFs were resuspended in the photoresins at 1 million cells mL^−1^. After 2P‐SL, the uncrosslinked bioresin was washed with 5× media changes prior to imaging. For the live/dead assay, FluoroBrite DMEM supplemented with 1:2000 CalceinAM (Invitrogen), 1:500 Propidium Iodide (PI, Fluka) was added. After 40 min, imaging was performed on a Leica SP8 microscope (Leica) equipped with a 25× objective. For “multistep/multicellular” of Figure 5C‐iii, NHDFs were previously incubated with CellTracker Green dye CMFDA (Invitrogen) and Cell Tracker Red dye CMTPX (Invitrogen) in serum‐free media at a working concentration of 10 µM. After 1 h in the incubator, green‐labeled and red‐labeled NHDFs were washed 3× with PBS. The two labeled‐NHDFs were resuspended at 1 million cells mL^−1^ in two separate photoresins (0.5% HA‐Mal and 0.79% PEG4SPC) and used for sequential 2P‐SL. After the printing of the first cylinder‐like structure with red‐labeled NHDFs, uncrosslinked bioresin was washed out with 5× media changes prior to adding the second green‐labeled NHDFs bioresin. After the printing of the external flower‐shape, uncrosslinked bioresin was washed out with 5× media changes prior imaging.

For constructs of Figure S9, Supporting Information, NG108‐15 neuronal cells were cultured in Neurobasal medium supplemented with 0.5 mm Glutamax and 2% B27, resuspended in the photoresin at 2 million cells mL^−1^ and printed as described above. At day 0, day 3, and day 6 of culture the gels were incubated in FluoroBrite DMEM supplemented with 1:2000 CalceinAM (Invitrogen) and imaged after 40 min using Leica SP8 confocal microscope (Leica) equipped with a 25× objective.

## Conflict of Interest

The authors declare no conflict of interest.

## Supporting information

Supporting information

## Data Availability

The data that support the findings of this study are openly available in ETH Research Collection at 10.3929/ethz‐b‐000568587, reference number 568587.
