# Supplementary material for: From Free‐Radical to Radical‐Free: A Paradigm Shift in Light‐Mediated Biofabrication
Source: Adv Sci (Weinh). 2023 Jan 25;10(8):2205302. doi: 10.1002/advs.202205302 (PMC10015869; doi:10.1002/advs.202205302)
Supplement: Supplementary file 1 — Supporting information [file ADVS-10-2205302-s001.pdf]

ADVANCED SCIENCE
Open Access

Supporting Information

for *Adv. Sci.*, DOI 10.1002/advs.202205302

From Free-Radical to Radical-Free: A Paradigm Shift in Light-Mediated Biofabrication

*Riccardo Rizzo, Nika Petelinšek, Angela Bonato and Marcy Zenobi-Wong**

Supporting Information

From Free-Radical to Radical-Free: A Paradigm Shift in Light-Mediated Biofabrication

*Riccardo Rizzo, Nika Petelinšek, Angela Bonato and Marcy Zenobi-Wong**

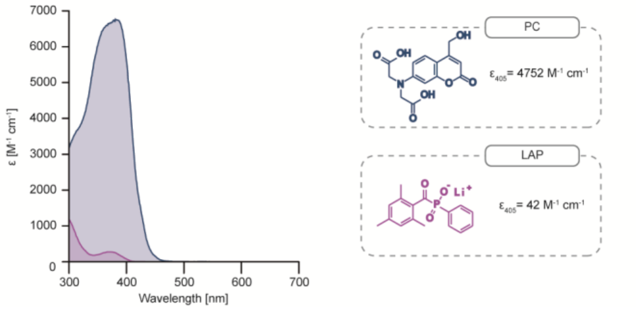

Figure S1. Absorption spectra of PC (blue) and LAP (magenta) with respective extinction coefficient (ε) values at 405 nm.

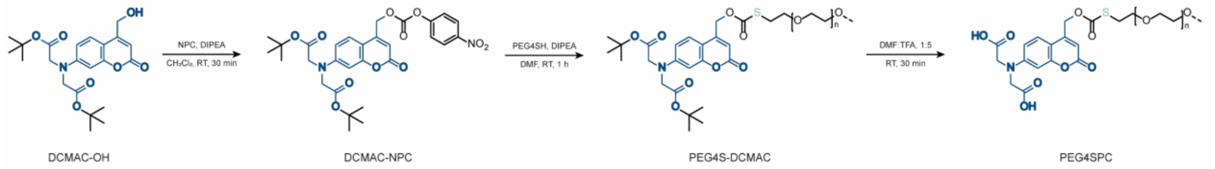

Figure S2. Final synthesis steps of PEG4SPC. The photocage 7-di-((tert-butyl-carboxy)methyl)amino 4-(hydroxymethyl)-coumarin (DCMAC-OH) was activated with 4-nitrophenyl chloroformate (NPC) and reacted with PEG4SH forming a thiocarbonate bond. Final product PEG4SPC was obtained with trifluoroacetic acid treatment (TFA).

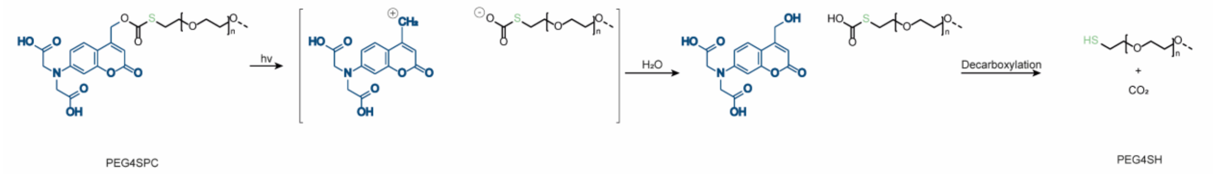

Figure S3. Uncaging process. Upon light absorption, PC (blue) is released from PEG4SPC with the formation of an intermediate coumarinylmethyl cation and thiocarbonate anion. PC cation reacts with solvent (H_2O) to generate the initial stable product (PC) while the unstable anion undergoes decarboxylation to give free thiol (PEG4SH).

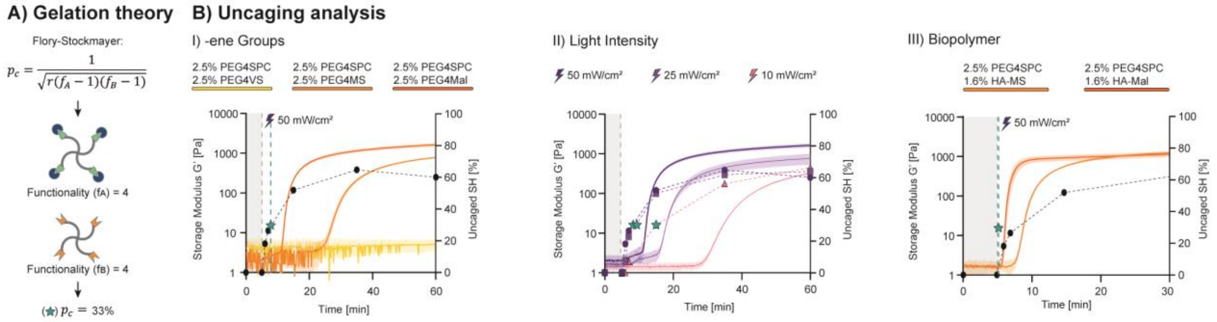

Figure S4. A) Flory-Stockmayer gelation theory reporting the critical gelation point (p_c) in relation to the molar ratio (r) of the two initial components and their functionalities (f_A and f_B). If considered as an ideal system, PEG4SPC and PEG4ene formulations have a p_c of 33%. Although far from being an ideal system, the formation of the gel based on PEG4SPC/PEGMal was the one that came closer to such theoretical estimate, suggesting a much faster reaction between uncaged PEG4SH and PEG4Mal when compared to PEG4MS and PEG4VS.

B) PC uncaging analysis (right y axis) performed under the same conditions used for photorheology (405 nm, gap: 200 µm, light intensity: 50, 25 or 10 mW cm^{2-1}). Photorheology curves shown in Figure 2B are overlapped (left y axis) to better visualize the relationship between uncaging and gelation kinetics of the various photoresin formulations. The theoretical gelation point is shown as a green star and dashed line. Interestingly, by varying the light intensity, the percentage of uncaged thiols showed significant difference in the first few minutes of irradiation (i.e., 18% for 50 mW cm^{2-1}, 7% for 25 mW cm^{2-1} and 0.1% for 10 mW cm^{2-1} after 1 minute irradiation) while reached a common plateau value ~60% after 1 hour probably due to the absorption of cleaved PCs that remain in the sample.

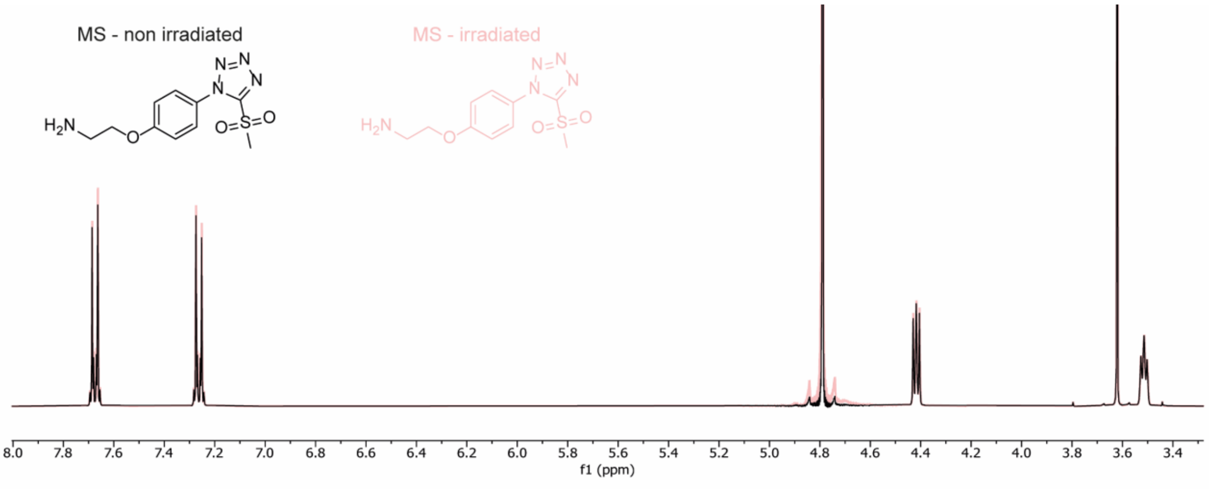

Figure S5. Photostability of MS verified with ^{1}H-NMR in D_2O. When irradiated with 405 nm light at 50 mW cm$^{2-1}$ for 30 min (pink trace), MS did not show signs of photodegradation (equivalent NMR spectrum compared to non-irradiated MS, black).

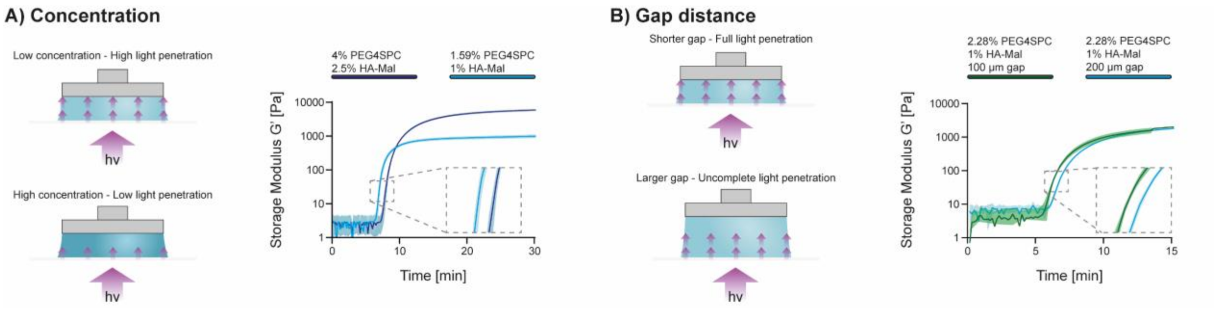

Figure S6. Impact of change in RF crosslinker concentration (A) and gap distance (B) during photorheology measurements. A) Because of the high extinction molar coefficient of PC at 405 nm, light does not efficiently penetrate throughout the higher concentrated photoresin (illustration on the left), therefore resulting in a slower crosslinking kinetics (right). However, when the total polymer concentration was increased, higher plateau storage modulus was found. B) By changing the gap distance between the rheometer probes, a similar effect can be observed as light does not efficiently penetrate through larger gaps (illustration left). Photorheology measurements showed, in fact, that when using the same photoresin, a faster crosslinking occurs with a shorter gap distance (right). Overall, these observations suggest that photorheology could be used to compare various photoresins when using the same measuring conditions and RF crosslinker concentration, but light absorption played a crucial role in the resulting crosslinking kinetics.

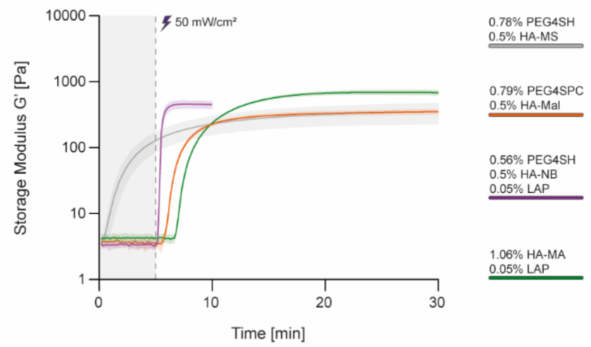

Figure S7. (Photo)crosslinking curves of photoresins adopted for ROS and Live/Dead assays (Figure 3) using the same irradiation conditions (50 mW cm$^{2-1}$, 0.5 mm gap). In addition, methacryloyl functionalized hyaluronic acid (HA-MA) with the same polymer content of HA-NB/PEG4SH was added as an example of chain-growth crosslinking kinetic.

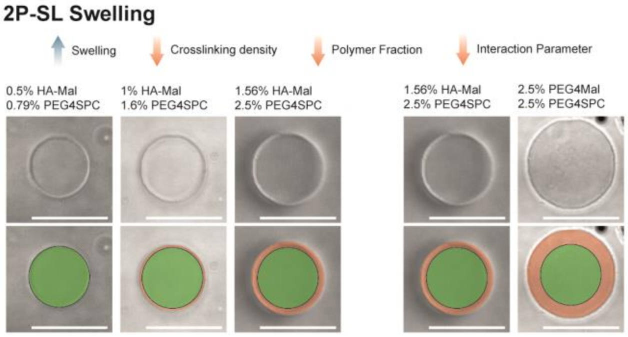

Figure S8. Swelling behaviour of various RF photoresins under optimal 2P-SL printing parameters (7.2 mW cm2-1, 3 scans). In green the correct size of the printed cylinders and in orange the swelled gel. Swelling is theoretically expected to increase with a reduction in crosslinking density, polymer fraction and interaction parameter (a small interaction parameter is correlated with a high enthalpic benefit of solvent infiltrating the network) (top). However, by increasing the polymer fraction (0.5, 1 and 1.56% HA-Mal) an increase of swelling was observed (left). As with an increase of HA-Mal there is also an increase of PEG4SPC concentration, the higher degree of swelling could be related to the PC removal. Although bearing hydrophilic carboxylic acids, the coumarin PC maintains a hydrophobic core that, upon uncaging, can determine a reduction in interaction parameter and a consequently favourable infiltration of the buffer solution. Also, when comparing HA-Mal and PEG4Mal formulations with the same PEG4SPC concentration, a significantly higher swelling is observed for the PEG-only photoresin. As the PEG4Mal/PEG4SPC photoresin has a higher polymer fraction and higher crosslinking density at full conversion (see Figure 2B), the increase in swelling can be correlated to the slower crosslinking kinetic of such photoresin.

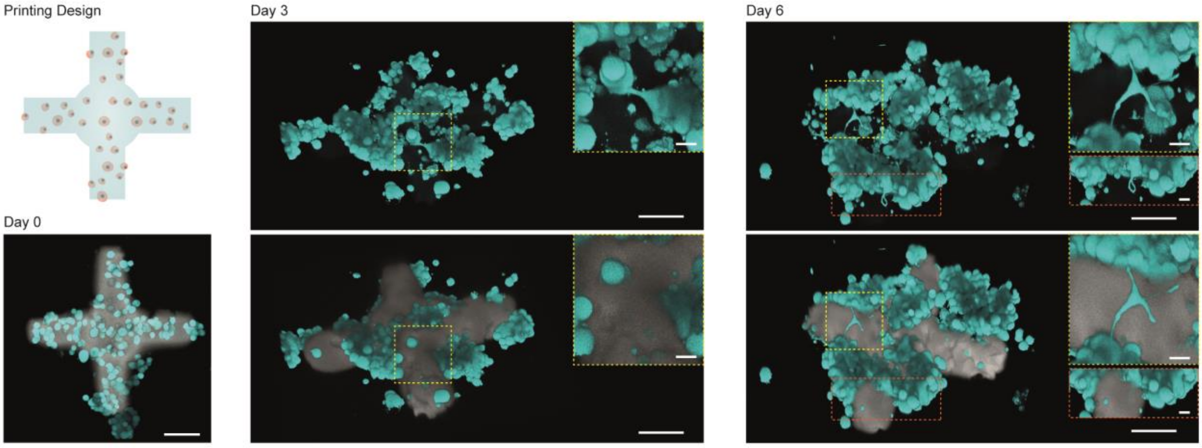

Figure S9. 2P-Biofabrication in the presence of neuronal cells (cyan) showing cell proliferation and neurite extensions within and on the printed constructs (grey) after 3 and 6 days in culture. Top and bottom rows show the same construct without (top) and with (bottom) printed gel. Scale bars: 100 µm (20 µm close ups).

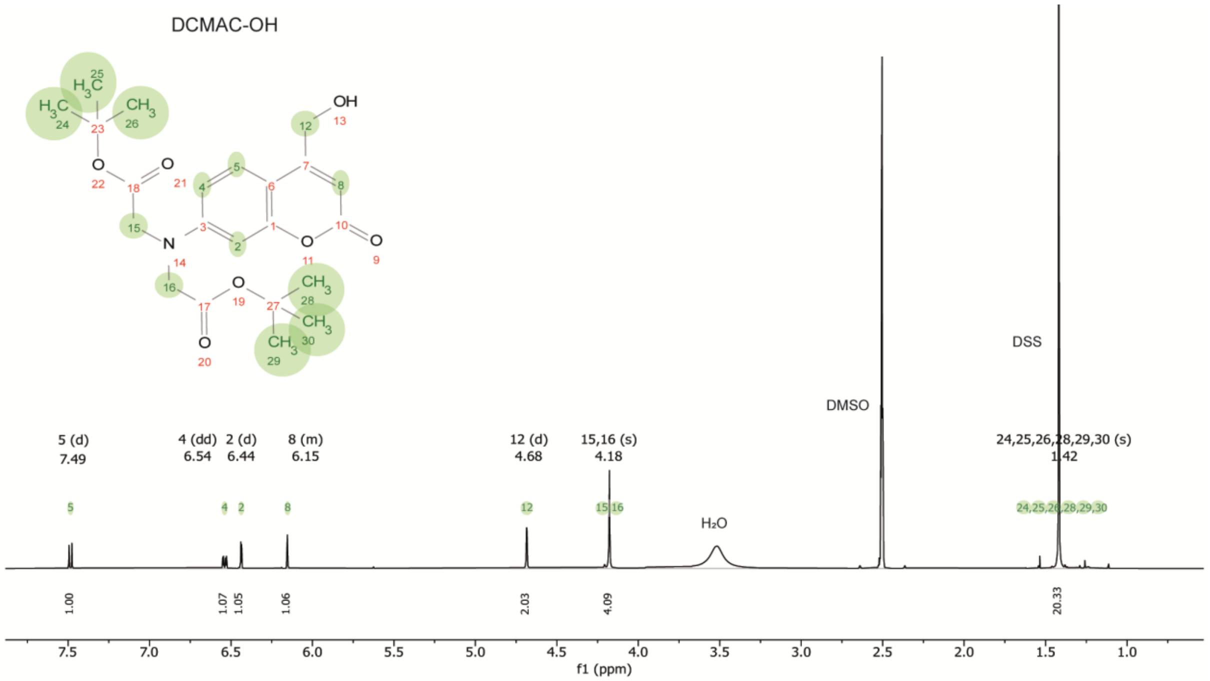

Figure S10. ^{1}H-NMR of DCMAC-OH in DMSO-d6. Assigned peaks are highlighted in green.

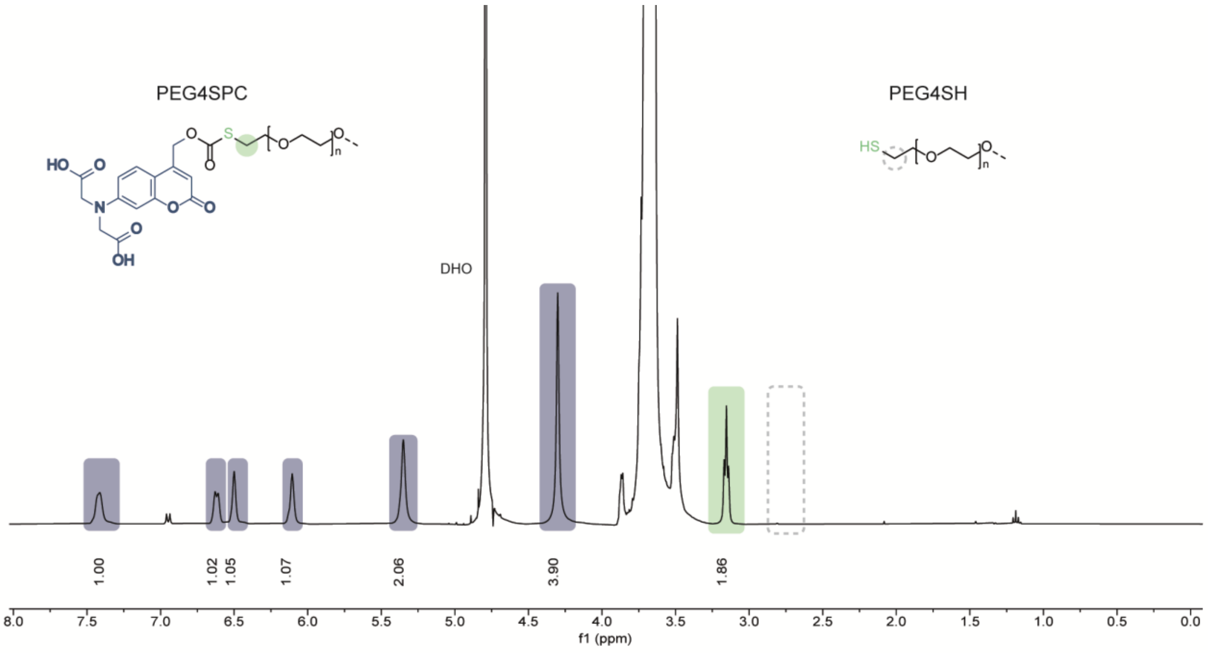

Figure S11. ^{1}H-NMR of PEG4SPC in D_2O. PC peaks (see Figure S7) are highlighted in blue, while terminal methylene group (2H, ~3.2 ppm) of the 4-arm-PEG is highlighted in green. Absence of terminal methylene group of PEG4SH (dashed line) confirmed complete caging of the thiols. As indicated in Figure 1C, the presence of the photocage shifts the terminal methylene peak of the PEG from ~2.7 to 3.2 ppm.

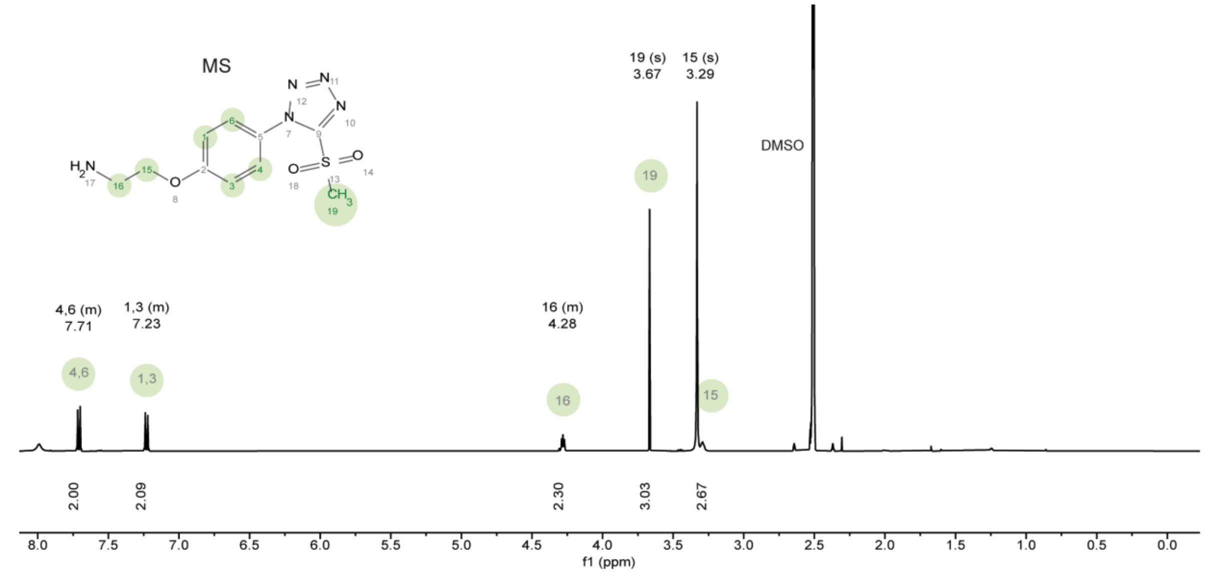

Figure S12. ^{1}H-NMR of MS in DMSO-d6. Assigned peaks are highlighted in green.

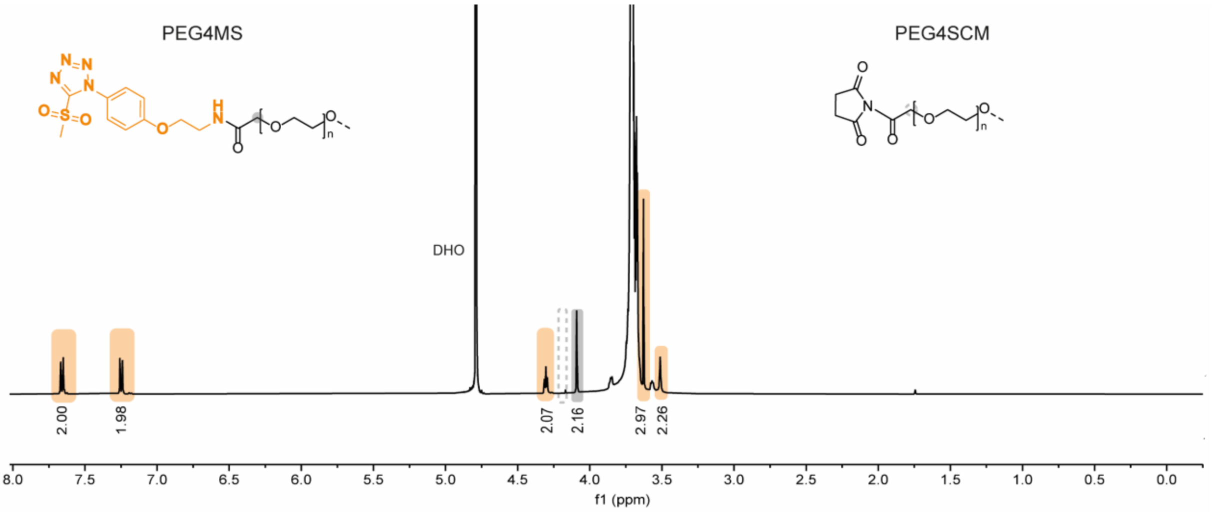

Figure S13. ^{1}H-NMR of PEG4MS in D_2O. MS peaks are highlighted in orange, while terminal methylene peak (2H, ~ 4.09 ppm) of the PEG chain is highlighted in grey. Absence of terminal methylene group of PEG4SCM (dashed line) confirmed completion of reaction. The presence of the MS moiety shifts the terminal methylene peak of the PEG from ~4.21 to 4.09 ppm.

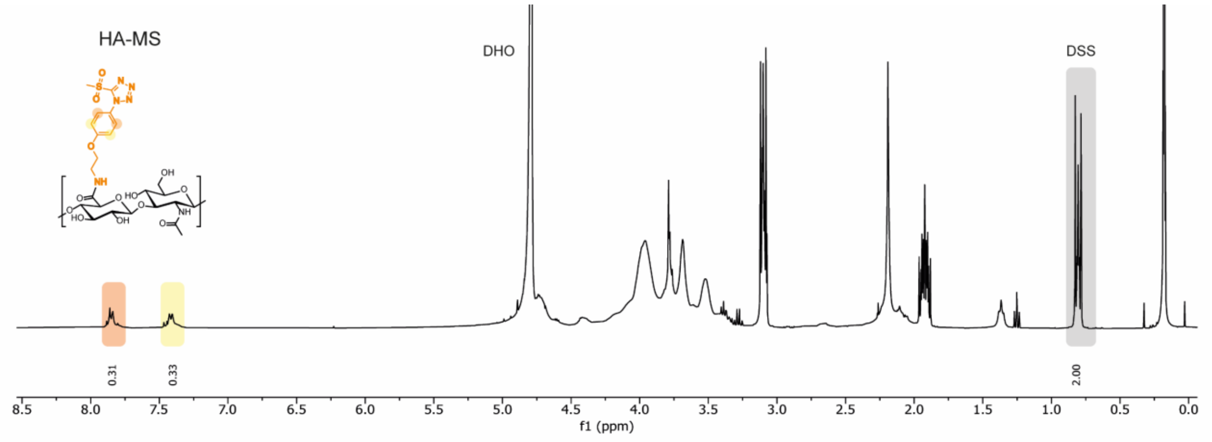

Figure S14. ^{1}H-NMR of HA-MS in D_2O. Internal standard 3-(trimethylsilyl)-1-propanesulfonic acid (DSS) is highlighted in grey (2H, ~0.75 ppm), and MS aromatic ring protons (4H, ~7.4 and 7.8 ppm) are highlighted in orange and yellow. These integrals were used to determine the HA-MS degree of substitution (DS: 22%).

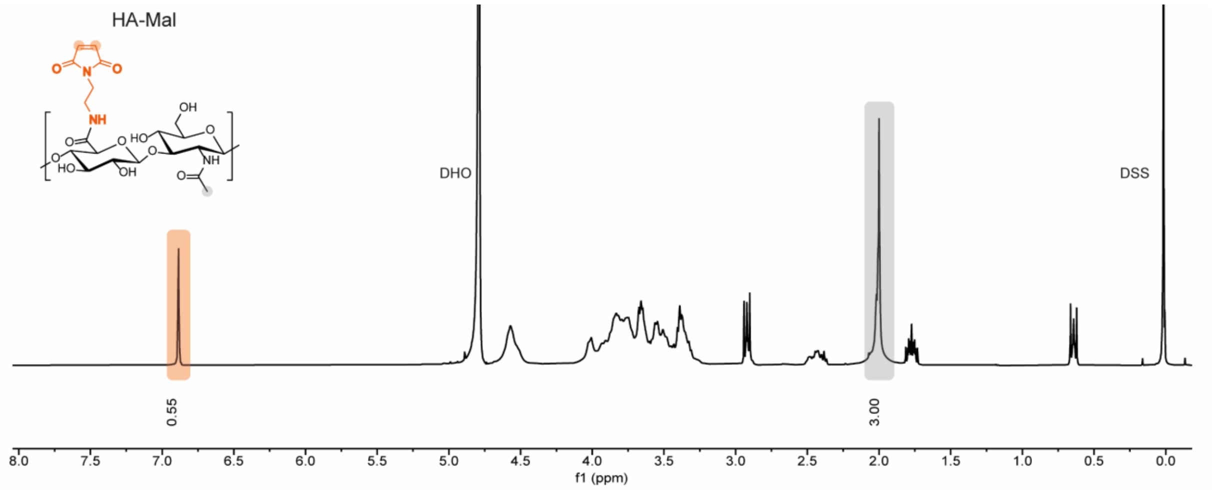

Figure S15. ^{1}H-NMR of HA-Mal in D_2O. Maleimide -ene protons (2H) are highlighted in orange (~7 ppm) and methyl protons of N-acetyl group are highlighted in grey (3H, ~2 ppm). These integrals were used to determine the HA-Mal degree of substitution (DS: 27.5%).

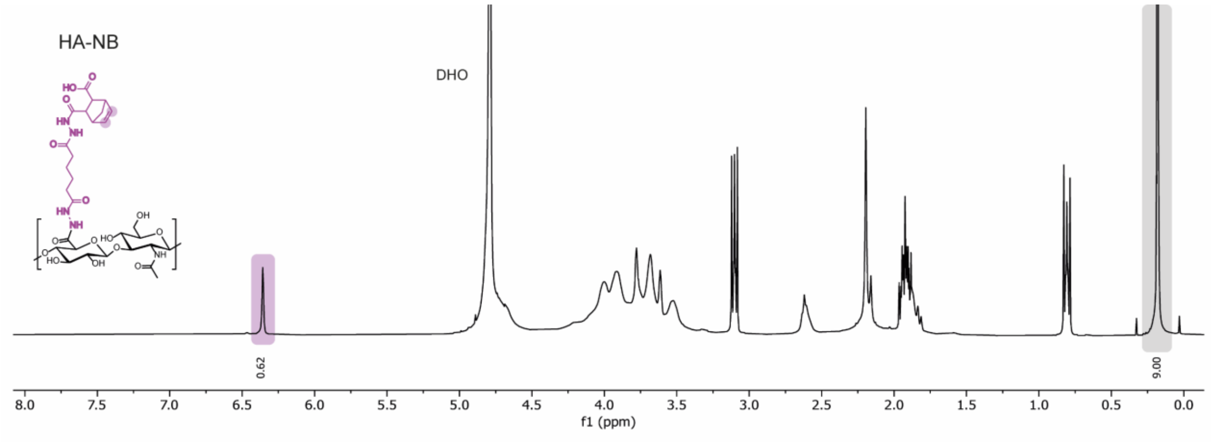

Figure S16. ^{1}H-NMR of HA-NB in D_2O. Norbornene -ene protons (2H) are highlighted in purple (~6.3 ppm) and internal standard 3-(trimethylsilyl)-1-propanesulfonic acid (DSS) is highlighted in grey (9H, ~0.2 ppm). These integrals were used to determine the HA-NB degree of substitution (DS: 18%).
